# Differential Use of Signal Peptides and Membrane Domains Is a Common Occurrence in the Protein Output of Transcriptional Units

**DOI:** 10.1371/journal.pgen.0020046

**Published:** 2006-04-28

**Authors:** Melissa J Davis, Kelly A Hanson, Francis Clark, J. Lynn Fink, Fasheng Zhang, Takeya Kasukawa, Chikatoshi Kai, Jun Kawai, Piero Carninci, Yoshihide Hayashizaki, Rohan D Teasdale

**Affiliations:** 1 Institute for Molecular Bioscience and ARC Centre in Bioinformatics, University of Queensland, St. Lucia, Queensland, Australia; 2 Advanced Computational Modeling Centre, University of Queensland, St. Lucia, Queensland, Australia; 3 Genome Exploration Research Group (Genome Network Project Core Group), RIKEN Genomic Sciences Center, RIKEN Yokohama Institute, Yokohama, Japan; 4 Genome Science Laboratory, Discovery Research Institute, RIKEN Wako Institute, Wako, Japan; The Jackson Laboratory, US; MRC-Harwell, UK; NHGRI-NIH, US; Lawrence Livermore National Laboratory, US; The Jackson Laboratory, US

## Abstract

Membrane organization describes the orientation of a protein with respect to the membrane and can be determined by the presence, or absence, and organization within the protein sequence of two features: endoplasmic reticulum signal peptides and alpha-helical transmembrane domains. These features allow protein sequences to be classified into one of five membrane organization categories: soluble intracellular proteins, soluble secreted proteins, type I membrane proteins, type II membrane proteins, and multi-spanning membrane proteins. Generation of protein isoforms with variable membrane organizations can change a protein's subcellular localization or association with the membrane. Application of MemO, a membrane organization annotation pipeline, to the FANTOM3 Isoform Protein Sequence mouse protein set revealed that within the 8,032 transcriptional units (TUs) with multiple protein isoforms, 573 had variation in their use of signal peptides, 1,527 had variation in their use of transmembrane domains, and 615 generated protein isoforms from distinct membrane organization classes. The mechanisms underlying these transcript variations were analyzed. While TUs were identified encoding all pairwise combinations of membrane organization categories, the most common was conversion of membrane proteins to soluble proteins. Observed within our high-confidence set were 156 TUs predicted to generate both extracellular soluble and membrane proteins, and 217 TUs generating both intracellular soluble and membrane proteins. The differential use of endoplasmic reticulum signal peptides and transmembrane domains is a common occurrence within the variable protein output of TUs. The generation of protein isoforms that are targeted to multiple subcellular locations represents a major functional consequence of transcript variation within the mouse transcriptome.

## Synopsis

Many genes produce only a single protein; however, others are known to produce a number of proteins with different functions in the cell. The function of a protein within the cell is influenced by its location; for example, proteins that are secreted can act as messengers, whereas proteins embedded in the membrane may act as receptors or channels. Features that determine the eventual location of a protein are found in the protein sequence. The authors identified two such features, the signal peptide that targets a protein for secretion, and the transmembrane domain that embeds a protein in the membrane, predicting their occurrence in mouse protein sequences. The authors then searched the entire mouse genome for genes that vary in the use of these features in protein isoforms. They found a large number of genes that produce proteins with variation in these features; for example, they identified genes producing proteins that are both secreted and intracellular, and genes producing proteins that are both membrane bound and soluble. This process is likely to be a major source of functional variation in the output of mammalian genes.

## Introduction

Recently, the murine transcriptome was redefined based on the sequences generated from the RIKEN FANTOM3 full-length mRNAs combined with the full-length mRNA sequences available in GenBank [1]. These transcripts were grouped into 43,539 transcriptional units (TUs), where a TU is a group of transcripts arising from a single genomic locus [1,2]. Of these TUs, 18,802 (38.6%) contained at least two variable spliced transcripts generated via alternative splicing and/or the use of alternative transcriptional initiation or termination sites. This level of transcript variation is consistent with previous studies that estimate that 30%–60% of mammalian genes are alternatively spliced [1,3], although there is evidence indicating that the true level of alternative splicing may be greater than 60% [4]. Significantly, because the FANTOM3 murine transcriptome is based on full-length cDNA transcripts and excludes partial or hypothetical transcripts, it becomes possible to systematically study the effects of transcript variation across an entire proteome—as opposed to elucidating the functional impact on proteins of transcript variation on a gene-by-gene basis [5]. Here we systematically search the mouse proteome for variation in protein features that define membrane organization.

Biological membranes partition eukaryotic cells into functional organelles and are themselves important functional components of the cell. The membrane organization of individual proteins represents the relationship of a protein to a membrane, that is, whether the protein is integral to a membrane, as opposed to secreted or cytoplasmic. Variation of membrane organization among the protein isoforms generated from the same TU will likely result in different subcellular localizations, and therefore functions, of those protein isoforms [5]. For example, a recent analysis of alternative splicing of 464 single pass transmembrane proteins proposed that 188 had a splice variant that created a soluble protein isoform [6].

We recently developed a membrane organization prediction pipeline, MemO (M. J. Davis, F. Zhang, Z. Yuan, and R. D. Teasdale, unpublished data), that classifies proteins based on the identification of alpha-helical transmembrane domains (TMDs) and the N-terminal endoplasmic reticulum signal peptide (SP) [7,8]. Briefly, to construct the MemO pipeline we first optimized the prediction of the SP and TMD features for eukaryotes using consensus approaches. We then incorporated a discrimination program to resolve conflicting predictions at the N-terminus [9] and established a set of annotation rules based on biological observations. The FANTOM3 Isoform Protein Sequence (IPS) sequences were clustered into TUs, and the application of MemO to these data enabled analysis of the way and extent that membrane organization changes between protein isoforms. This revealed candidate genes (or TUs) where transcript variation serves as a mechanism for regulating protein functionality by altering the membrane organization of the protein isoforms generated. Further analysis revealed common mechanisms of transcript variation used to modulate the inclusion of both signal peptides and transmembrane domains.

## Results

### Membrane Organization Classification

The membrane organization annotation pipeline MemO was applied to the IPS mouse protein set created by the RIKEN FANTOM3 project [1]. Before application of MemO, protein sequences derived from protein-coding transcripts were filtered to remove putative non-full-length sequences. Protein sequences without an initial methionine or with coding sequences clearly annotated as truncated were removed. Protein sequences shorter than 30 residues long or including nonstandard amino acid symbols were also removed. The remaining 33,451 IPS protein sequences were annotated using the pipeline. Transcript sequences had previously been clustered into 19,538 TUs [1,2]. In the IPS dataset, 5,116 protein sequences (15.3%) were predicted to contain signal peptides and 8,238 protein sequences (24.6%) were predicted to contain TMDs. Of 2,029 sequences with feature prediction conflicts in the N-terminal sequence, 1,638 were resolved as signal peptides and 391 as transmembrane domains. The inclusion of multiple protein isoforms within the IPS dataset did not alter the proportional distribution of these protein features when compared with the results observed in previously analyzed representative proteins sets from mouse and other species [10]. A summary of the annotation of the IPS dataset into the five membrane organization classes is provided in Table 1.

**Table 1:**
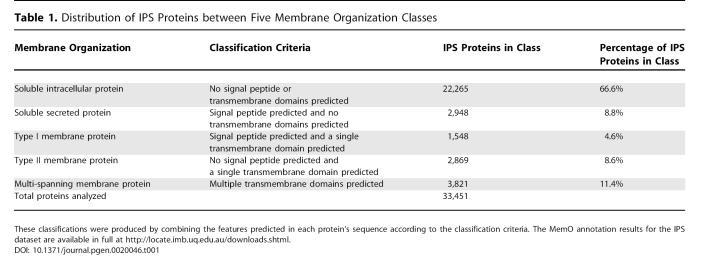
Distribution of IPS Proteins between Five Membrane Organization Classes

Within the IPS set, there were 8,032 TUs that contained two or more nonidentical protein products, representing a total of 21,913 protein-coding transcripts. To determine the impact of transcript variation on membrane organization within TUs, we analyzed the variation of each region of the transcript encoding the predicted features. As 5,036 multi-protein TUs did not contain either feature they were excluded from the analysis. The resulting set of 2,996 multi-protein TUs contained 8,157 protein-coding transcripts. Variable features are those that do not share the same genomic region while ubiquitous features are present in all transcripts of the TU and use the same genomic region to encode the feature. Each TU was then examined to discover the degree of variability in the genomic location of SPs and TMDs.

### Feature Variation: Signal Peptides

A total of 1,475 TUs contained one or more transcripts where the protein product was predicted to encode a SP, of which 760 (51.5%) used a SP arising from one genomic location in all transcripts produced from the TU. An additional 142 TUs (9.6%) lacked sufficient data in the genomic alignment of transcripts to determine the pattern of SP usage. The remaining 573 TUs (38.8%) showed some variation in SP usage among transcripts. We refer to this set as the variable signal peptide (VarSP) set (TUs with gene identifiers are listed in Table S1).

**Figure 1:**
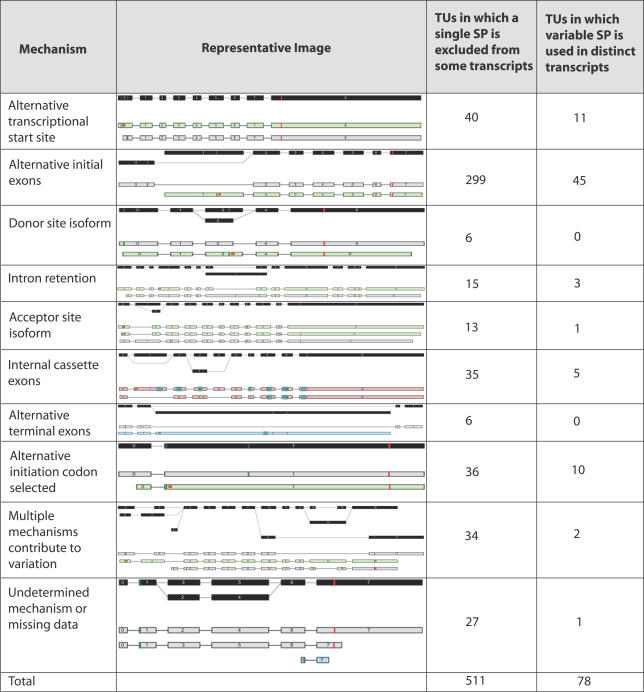
Variation of Signal Peptide by Transcript Variation and Alternative Splicing Sixteen TUs are represented in both categories. These TUs contain multiple transcripts using signal peptide coding regions generated from distinct regions of the genome while alternative transcripts within the same TU exclude these signal peptide coding regions altogether. Thin green and red bars across exons represent the location of the start and stop codons respectively. An orange dot following the start codon represents the presence of N-terminal signal peptide, while green blocks show the genomic localization of the predicted transmembrane domain features within exons.

From the VarSP set, 511 TUs were found to have a SP arising from one genomic location that was used in some transcripts but was absent in others. We first examined the mechanisms of signal peptide exclusion in these TUs (Figure 1). The most common mechanism of variation was the use of an alternative initial exon that did not encode a SP (58.5%). The use of alternative transcriptional start sites (7.8%) and internal cassette exons (6.8%) was also observed. Signal peptide exclusion was also caused by intron retention, donor site isoforms, acceptor site isoforms, and alternative terminal exons (see Figure 1). There were 36 cases where the cause of signal peptide exclusion appeared to be the selection of an alternative initiation codon within the transcript that resulted in the exclusion from the coding sequence of the region of transcript encoding a signal peptide in other isoforms. No variation was observed in the splicing of the transcripts from these TUs, so the selections of alternative initiation codons most likely represent incorrect annotations. Alternatively, they may be caused by polymorphisms within the genome rather than transcript variation.

A second type of variation observed in the VarSP set was signal peptide replacement, where some protein-coding transcripts used a SP arising from one genomic location, while other transcripts used a SP encoded by an alternative genomic region. From the VarSP set, 78 TUs were observed with this type of variation (Figure 1). Alternative initial exon usage was the most common mechanism for replacing one signal peptide with another (57.7%). One striking example, TU71446, encoded 48 isoforms of protocadherin using 34 different signal peptides, each encoded by its own initial exon. Variation of the signal peptide through alternative transcriptional start site usage was also observed (14.1%).

These data collectively indicate that for TUs with multiple protein products and predicted signal peptide features, approximately 40% show variation of the signal peptide through transcript variation, while over half contain a signal peptide that does not vary, arising from one genomic location in all transcripts.

### Feature Variation: Transmembrane Domains

Within the multi-protein, feature-positive TU set described above, 2,329 TUs contained one or more transcripts where the protein product was predicted to encode a TMD. A total of 885 TUs (38.0%) were found to have the same number of transmembrane domains in all transcripts produced. In the vast majority of these (802), all transcripts used the same region of the genome to encode the TMD, however 83 TUs, while maintaining the same number of TMDs, used alternative TMDs encoded by different regions of the genome, or contained genomic regions not predicted as TMDs in all transcripts. In the remaining 62.0% of the TMD-positive set, the number of TMDs in protein isoforms varied. Together, a total of 1,527 TUs, representing ~66% of the TMD-positive set, were found to have variable transmembrane domain usage; these are collectively referred to as the variable TMD (VarTM) set (TUs with gene identifiers are listed in Table S1).

**Figure 2:**
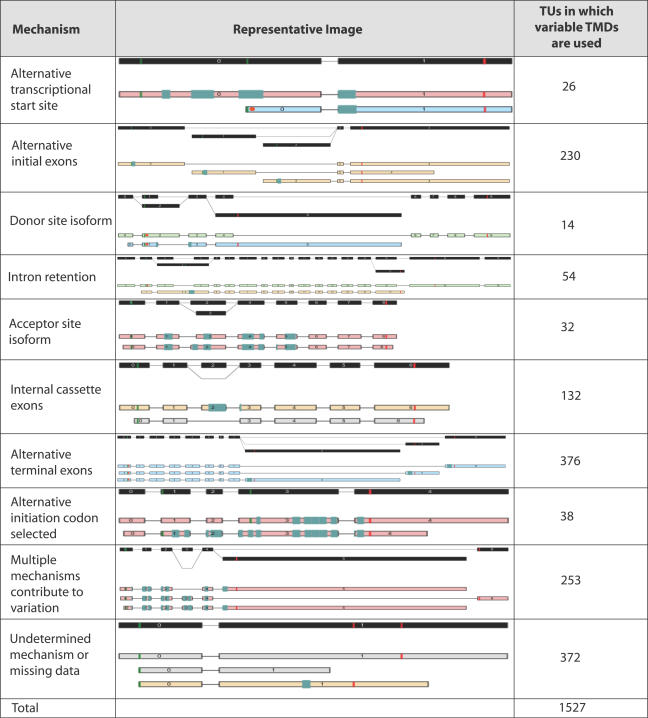
Variation of TMDs by Transcript Variation and Alternative Splicing

The VarTM set was examined to determine the mechanisms used to vary transmembrane domain usage. Around 45% of TUs in the VarTM set contained protein-coding transcripts predicted to contain a single TMD lacking in other protein-coding transcripts. For the vast majority of these TUs (~98%), the TMD-positive transcripts used the same feature; however, the remaining 17 contained additional variation of the TMD feature by use of an alternative genomic region to encode it. Most commonly, these TUs used two mutually exclusive alternative regions to encode the TMD, although the use of up to four alternative mutually exclusive regions was observed. Variability was also observed in the number of TMDs predicted in each protein-coding transcript. Nearly half of the VarTM set contained at least one transcript predicted to code a multi-spanning membrane protein as well as other transcripts predicted to have different numbers of the TMD feature. The range of major mechanisms producing variation of the predicted TMDs was broader than that observed for signal peptides (see Figure 2). Alternative initial exons, internal cassette exons, and alternative terminal exons were the most common mechanisms observed to generate transcripts with variable TMDs. Also, combinations of mechanisms were frequently observed to generate variation.

These data collectively indicate that for TUs with multiple protein products and predicted transmembrane domain features, 65% undergo some kind of variation of the transmembrane domain through transcript variation. The remaining 35% contain a single set of ubiquitously used transmembrane domains.

### Alternative Splicing in the VarSP and VarTM Sets

To classify the alternative splicing events within TUs, independent of the individual protein features, we applied a modified computational classification scheme developed by Clark and Thanaraj [11]. Within the 8,032 TUs with two or more nonidentical protein products, 71 TUs did not have complete genomic mappings available and were excluded from this analysis. The results are presented in Table 2. A chi-square statistical test was applied to the VarSP and VarTM sets in order to determine if the distribution of the alternatively spliced events observed in those sets was significantly different to that seen in the global multi-protein TU set. Both the VarSP and VarTM sets had significantly different patterns of alternative splicing events compared to the global set, with *p* < 0.0001 for both comparisons. This indicates that the TUs that make up the VarSP and VarTM sets represent different populations with respect to their alternative splicing properties. We compared the proportions of alternative splicing events observed in each set to those observed in the global multi-protein TU set. The use of cassette exons was the only overrepresented alternative splicing event within both the VarSP (1.34-fold) and VarTM (1.26-fold) sets. Intron retention (1.24-fold) and transcriptional start sites (1.11-fold) were overrepresented in the VarSP set, while other events showed a proportional variation of less than 10%. These overrepresented alternative splicing events, based on all transcript variation, corresponded to the major mechanisms of transcript variation identified for the individual features (see Figures 1 and 2).

**Table 2:**
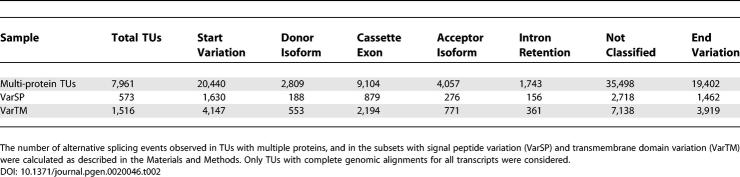
Comparison of Events Causing Transcriptional Variation

### Variable Membrane Organization

**Figure 3:**
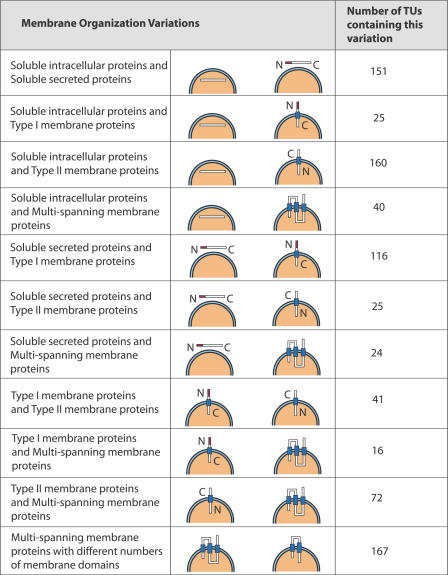
Categories of Membrane Organization Observed in the 782 High-Confidence Variable TUs In total, 753 TUs occurred in two different membrane organization classes, while 29 TU's occurred in more than two membrane organization classes, and are present in a number of variation categories.

Within the set of 2,996 multi-protein, feature-positive TUs, 1,380 were identified with variation in the predicted membrane organization categories as outlined in Table 1. In addition, 319 TUs classified as multi-spanning membrane proteins were identified with variation in the number of transmembrane domains. This resulted in a set of 1,699 TUs with variable membrane organization (Table S1), which were manually reviewed. First, we ensured that there was some overlap in protein sequence between isoforms showing divergent membrane organization. Second, transcripts were identified in this set that had exons with identical genomic coordinates but were annotated with different protein-coding sequences. Frequently, several transcripts from the same TU were observed to contain identically located genomic exons, but had a small number of base pair inconsistencies when aligned to the genome. These inconsistencies may represent sequencing errors or mouse strain polymorphisms, and could result in frame shifts in the corresponding protein sequence or disruption of the coding sequence region, causing the observed variation of the membrane organization features. Third, annotation of membrane organization features by MemO was influenced by the presence of variable protein sequences outside the sequence encoding the feature. The result of this sometimes included splitting of a single predicted transmembrane domain into two smaller predicted domains, thus altering the count of TMDs. Finally, transcripts that showed no evidence of splice variation and may represent truncated versions of full-length transcripts were critically evaluated for inclusion in a high-confidence set. Only transcripts that contained independent support for the alternative transcriptional initiation and termination sites were included, thus not all transcripts present in each TU were included in the high-confidence set. Supporting evidence for the alternative transcriptional start and end sites was evaluated using the Genomic Elements Viewer (http://fantom32p.gsc.riken.jp/gev-f3/gbrowse/mm5). This database includes all 5′ and 3′ boundaries of the 181,047 independent transcripts within the mouse transcriptome and their frequency of use based on evidence from full-length cDNA sequencing and cap analysis gene expression (CAGE) and related methodologies [1]. The resulting set of 782 TUs that passed this manual curation process will subsequently be referred to as the variable membrane organization (VarMO) set (Figure 3; Table S1).

Within the VarMO set, 586 TUs had two different membrane organization classifications annotated, while 29 TUs had more than two membrane organization classifications observed for the encoded protein isoforms. The remaining 167 TUs possessed transcripts ubiquitously classified as multi-spanning membrane proteins, but with variable numbers of transmembrane domains.

While all pairwise combinations of membrane organization classes are observed within the VarMO set, several combinations were more frequent. For example, 376 TUs contained at least one protein-coding transcript encoding a soluble intracellular protein. Other protein-coding transcripts in these TUs were mainly predicted to contain either type II membrane proteins (46.8%) or soluble secreted proteins (37.0%). Furthermore, for TUs containing a protein-coding transcript predicted to be a soluble secreted protein, a bias towards soluble intracellular proteins (46.1%) and type I membrane proteins (27.9%) was observed.

The VarMO set was compared to multi-protein TUs in the IPS set, and the general properties of both sets were found to be similar. For example, VarMO contains 782 TUs with an average of 3.0 transcripts per TU, and the whole multi-protein TU set contains 8,032 TUs with an average of 2.7 transcripts per TU. The proportion of membrane organization classes in the IPS set and the subset of TUs with multiple proteins varied from those seen in the VarMO set. For example, soluble secreted proteins are present in only 11%–12% of TUs in the larger sets, but are present in 38% of TUs from the VarMO set, while 5%–7% of TUs in the larger sets contain type I membrane proteins, compared to 22% of TUs from the VarMO set (see Tables 3 and S1).

**Table 3:**
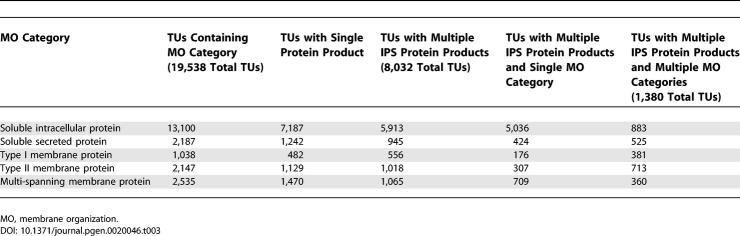
Analysis of Membrane Organization Variation at the Level of TUs for 33,451 Protein Sequences Belonging to 19,538 TUs

To better understand the composition and biological nature of these data subsets, we generated a set of Gene Ontology (GO) terms significantly overrepresented in each combination of membrane organization classes. We looked for terms overrepresented in the variable categories when compared with sets of each individual membrane organization taken from the IPS set as a whole (Table S2). Individual TUs encoding type I membrane proteins, particularly transmembrane kinase receptors associated with signal transduction, cell communication, and cell adhesion, frequently generated soluble protein products encoding the extracellular ligand-binding domains or the intracellular kinase/signaling domains. In addition, truncated membrane-anchored variants (i.e., type II membrane proteins) of these type I transmembrane receptors were also generated. These variant protein products will clearly influence the outcomes of cell signaling events. This highlights the importance of determining which protein variant is generated when examining the role these proteins play in various biological signaling processes. Likewise, TUs encoding multi-spanning membrane proteins, particularly those associated with ion transport and receptor activities also generate soluble intracellular and extracellular protein products. Numerous TUs encoding soluble enzymes associated with cellular catabolism generate both intracellular and extracellular variants. Critically, the identification of sets of TUs associated with particular biological processes and containing protein isoforms from multiple membrane organization classes validates the selectivity of the computational approach used to identify them.

## Discussion

Changes to the membrane organization of individual proteins will modulate the function of a protein by altering the cellular environment with which it is associated. Post-translational proteolysis, including regulated intercellular proteolysis [12] and ectodomain shedding [13], represents one mechanism used to achieve variation in membrane organization. We have reported here that variable transcript output from the same gene or TU is another mechanism commonly used to vary the membrane organization of proteins. Previously, others have used computational approaches for examining genes that highlighted specific aspects of this variation, including the identification of soluble proteins from single-spanning membrane proteins [6], the study of removal of transmembrane domains and signal peptides in theoretical proteins re-created from public cDNA sequences [14], and the identification of putative soluble variants of membrane proteins through annotations in UniProt [15]. Our study represents a more comprehensive approach that systematically analyzed the entire mouse transcriptome, as defined by Carninci et al. [1].

Our analysis of the mouse transcriptome identified 8,032 TUs that encoded multiple protein isoforms. A question raised by the high numbers of mammalian genes undergoing alternative splicing is the extent to which this splicing results in functional variation of the protein products. By focusing on protein variants that have different membrane organizations, we identified a set of genes where alternative splicing has a direct functional consequence, as changes in membrane organization are frequently associated with changes in subcellular localization, or availability of the protein for protein–protein interaction [5].

From 19,538 TUs, we identified 2,996 (15%) that contained multiple proteins and are predicted to contain at least one signal peptide or transmembrane domain. Of these, 1,475 (49%) contained at least one predicted signal peptide, while 2,329 (78%) contained at least one transmembrane domain. These proportions correspond with 8% and 12%, respectively, of the total number of protein-coding TUs in our filtered IPS set. Within the set of 1,475 TUs encoding signal peptides, 39% were found to vary their use of signal peptides. In the vast majority of cases this variation caused the exclusion of the predicted signal peptide from one or more protein products. In other cases, variation involved the replacement of one signal peptide with another encoded by a different genomic region. Within the set of 2,329 TUs with multiple proteins and one or more transmembrane domains, only 802 (34%) were found to contain identical complements of ubiquitously used transmembrane domains in all protein products, while 35% showed variations that would result in the generation of a variant soluble form of the TMD-containing protein. The remainder produced proteins with a variable complement of transmembrane domains.

Characterization of the mechanisms used to generate the transcript variation relative to the predicted feature revealed that a number of common strategies were utilized. Signal peptides are only present at the N-terminus of a protein's coding sequence and are, therefore, typically encoded within the first or second exon. Consistent with this, we observed a predominant use of alternative initial exons or alternative transcriptional initiation sites in cases of SP variation. In contrast, the mechanisms used to vary individual transmembrane domains were more diverse. Alternative initial exons, internal cassette exons, and alternative terminal exons were all frequently exploited to generate transcripts with variable numbers of transmembrane domains. Variations of the exon boundaries (donor site isoform, acceptor site isoform, and intron retention) were also observed for both signal peptides and transmembrane domains but at a lower frequency than the mechanisms exploiting mutually exclusive regions of the genome**.**

The datasets used and generated in this analysis are of a high quality. The IPS set has been systematically reviewed [1,16], and protein sequences were further filtered prior to submission to the MemO pipeline. This two-stage removal of any apparently suspect non-full-length protein sequences provides increased confidence that the events described here represent true biological events rather than artifacts created by incomplete transcripts. Other problems that can occur with expressed sequence tag clustering and/or mapping to the genome [3] are also avoided. Furthermore, for the high-confidence VarMO set, we established strict, conservative criteria for inclusion, including manual curation of this set.

A criticism sometimes made of computational approaches for the identification of alternative splicing has been that these approaches do not place predicted events in a biological context in the same way that experimentally characterized gene-specific alternative splicing events are framed [17]. In this computational analysis we provide biological context to the observed events by focusing on genes with variable membrane organization, rather than cataloging alternative splicing events in the whole IPS set. That is, the VarMO set contains TUs that produce protein isoforms that are annotated to be in more than one membrane organization category. We have focused our analysis on this high-confidence set and because of our conservative criteria for inclusion, we are likely to be underestimating the number of TUs with variable membrane organization.

Critically, in this computational study we identified a number of experimentally validated genes where alternative splicing is known to cause variation in the membrane organization of encoded protein isoforms. Across the observed combinations, these include soluble intracellular proteins and soluble secreted proteins—sialic 9-O-acetylesterase [18]; soluble intracellular proteins and type I membrane proteins—protein tyrosine phosphatase [19]; soluble intracellular proteins and type II membrane proteins—CUTL1 [20], protein tyrosine phosphatase [21], and bcl-x [22]; soluble intracellular proteins and multi-spanning membrane proteins—Lmbr1 [23]; soluble secreted proteins and type I membrane proteins—IL-4 receptor [24], CD40 [25], inhibin binding protein [26], neuropilins 1 and 2 [27], epidermal growth factor receptor [28], Flt-1 [29], granulocyte-macrophage colony stimulating factor [30], 4–1BB [31], Flt-3 ligand [32], Fit-1 [33], IL-2 receptor [34], and the leptin receptor [35]; soluble secreted proteins and multi-spanning membrane proteins—thyroid stimulating hormone receptor [36]; type I membrane proteins and type II membrane proteins—protein tyrosine phosphatase Ptprr [37]; multi-spanning membrane proteins with different numbers of domains—mercurial-insensitive water channel 3 [38], cystic fibrosis transmembrane conductance regulator [39], porcupine-D [40], X transporter protein 2 [41], urea transporter isoform UT-A1 [42], and Atp2a2 [43].

Within the VarMO set all possible combinations of membrane organization class switching were observed. From most common to least, the major membrane organization class variations observed were as follows: multi-spanning membrane proteins with different numbers of domains soluble intracellular proteins and type II membrane proteins, soluble intracellular proteins and soluble secreted proteins soluble secreted proteins and type I membrane proteins, and type II membrane proteins and multi-spanning membrane proteins. While to date the majority of experimentally validated examples are within the sets (1) soluble secreted proteins and type I membrane proteins and (2) multi-spanning membrane proteins with different numbers of domains, support does exist for the occurrence of the other classes.

Based on our analysis of the 8,032 TUs with variable protein-coding transcripts, we conservatively estimate that approximately 10% contain differentially encoded signal peptides and/or transmembrane domains. This indicates that variation of membrane organization is a major outcome of alternative splicing and/or transcript variation. All of these observations have been incorporated into the LOCATE database (http://locate.imb.uq.edu.au), which also provides further biological context for these proteins through integration with domain predictions, subcellular localization data collected from the literature and high-throughput experiments, and links to other database resources [44].

## Materials and Methods

### Datasets.

The IPS dataset created by the RIKEN FANTOM3 Consortium from novel and public protein-coding transcripts [1] was the base dataset for this work. The sequences in the IPS set were generated exclusively from direct sequencing of full-length transcripts and do not include any hypothetical transcripts. The data have been clustered into TUs, that is, groups of transcripts arising from a single genomic locus, defined as sharing at least one nucleotide having the same genomic location and orientation [1,2]. Protein isoforms generated from each TU are available, and all sequences in the IPS set have some variation at the protein level. This dataset is accompanied by the genomic alignments of the spliced transcripts. This dataset is available at ftp://fantom.gsc.riken.jp/RTPS/fantom3_mouse/primary_rtps/IP [1].

### Membrane organization annotation.

Membrane organization of proteins within the IPS dataset was annotated using the pipeline method MemO (M. J. Davis, F. Zhang, Z. Yuan, and R. D. Teasdale, unpublished data), which classifies proteins into five major classes of membrane organization: soluble intracellular proteins, soluble secreted proteins, type I membrane proteins, type II membrane proteins, and multi-spanning membrane proteins. MemO generates predictions of two main features, signal peptides and transmembrane domains, using consensus methods to achieve greater accuracy [45]. Five methods contribute to the consensus prediction of transmembrane domains: SVMtm [46], TMHMM [47], HMMTOP [48], Memsat [49], and DAS [50]. In order to be annotated as a transmembrane domain, regions of protein sequence must have positive transmembrane domain predictions from at least three of the predictors used. Regions shorter than five residues are discarded, and regions separated by gaps of less than four residues are joined into a single region. Three methods contribute to the prediction of signal peptides: SPScan [51] and the two Signal P V.2 methods of neural-network-based [52] and hidden-Markov-model-based [53] prediction. Conflicting predictions in the first 45 residues are resolved using a previously published method [9]. Features are predicted at the protein sequence level.

### Analysis of transcript variation.

Predicted protein features (SPs and TMDs) were mapped to genomic coordinates. Genome alignments of the transcripts were used to generate exon-splicing graphs for each TU [54]. These graphs are presented within LOCATE [44] at http://locate.imb.uq.edu.au, and links to these graphs are presented in Table S1. The splicing graphs shown in LOCATE have been generated from the primary data, and do not reflect confirmed transcript variations in our high-confidence VarMO set. These graphs were classified using a previously described system [54] (http://proline.bic.nus.edu.sg/dedb/methodology.html).

Alternative splicing events within a TU were identified and classified computationally. Within a given TU, observed exons were compared with observed introns, and any overlap was taken to indicate alternative splicing. An exon encoded entirely within an intron was labeled as a cassette exon. Exons were also compared to other exons; if two exons shared a donor splice site but differed at the acceptor splice site, an acceptor site isoform was recorded—and vice versa for a donor site isoform. In the case that two exons overlapped but differed at both ends, those exons were recorded as alternatively spliced but not classified. Intron retention was recorded when an intron was entirely contained within an exon. Variation in the transcriptional start and end points of the transcript was also recorded. All exons identified as alternatively spliced were recorded according to genomic coordinates and the category of splice variation observed.

### GO analysis.

Mouse Genome Informatics accession numbers [55,56] associated with the IPS set were used to conduct GO analysis. The GOstat application, available at http://gostat.wehi.edu.au [57], was used to retrieve GO terms and to determine which were significantly over or under represented in the datasets. Across the membrane organization categories, the numbers of TUs that had GO annotations are as follows: 7,088 TUs containing soluble intracellular proteins, 1,494 TUs containing soluble secreted proteins, 728 TUs containing type I membrane proteins, 1,150 TUs containing type II membrane proteins, and 1,685 TUs containing multi-spanning membrane proteins. Of the TUs within the VarMO set, 715 had GO annotations.

## Supporting Information

Table S1 Results for 8,032 Multi-Protein TUs from the IPS Set Gene names, Mouse Genome Informatics identifiers, and EntrezGene identifiers are presented for the listed TUs. GenBank accession numbers, or, where these are not available, RIKEN accession numbers, for the transcripts clustered in each TU are also listed. Presence of each TU in the VarSP, VarTM, and VarMO sets is indicated, as are the classes of membrane organization predicted in each TU. Links to the LOCATE database (http://locate.imb.uq.edu.au) and the splicing graphs generated for each TU are also provided.
(5.7 MB XLS)

Table S2 GO Terms Overrepresented in VarMO Lists were created for the variable sets corresponding to ten types of membrane organization variation present in the VarMO set of TUs. These lists were each compared with the two membrane organization class sets corresponding to each individual category observed in the variable type. For these ten comparisons, *p* = 0.01 was used.
(16 KB PDF)

## Abbreviations

GO: Gene Ontology
IPS: Isoform Protein Sequence
SP: N-terminal endoplasmic reticulum signal peptide
TMD: alpha-helical transmembrane domain
TU: transcriptional unit
VarMO: variable membrane organization
VarSP: variable signal peptide
VarTM: variable alpha-helical transmembrane domain
