# Supplementary material for: Differential Use of Signal Peptides and Membrane Domains Is a Common Occurrence in the Protein Output of Transcriptional Units
Source: PLoS Genet. 2006 Apr 28;2(4):e46. doi: 10.1371/journal.pgen.0020046 (PMC1449889; doi:10.1371/journal.pgen.0020046)
Supplement: Table S2 — Lists were created for the variable sets corresponding to ten types of membrane organization variation present in the VarMO set of TUs. These lists were each compared with the two membrane organization class sets corresponding to each individual category observed in the variable type. For these ten comparisons, p = 0.01 was used. (16 KB PDF) [file pgen.0020046.st002.pdf]

Variable classes of protein orientation with respect to the membrane	Comparative category	Over-represented Gene Ontology terms
Soluble intracellular and soluble secreted proteins	Soluble intracellular protein	Cytolysis (GO:0019835); Cellular protein catabolism (GO:0044257); Receptor binding (GO:0005102); Transforming growth factor beta receptor binding (GO:0005160); Serine-type peptidase activity (GO:0008236); Extracellular matrix structural constituent conferring tensile strength (GO:0030020); Frizzled-2 signaling pathway (GO:0007223); Glial cell differentiation (GO:0010001); Serine-type endopeptidase activity (GO:0004252); Endopeptidase activity (GO:0004175); Extracellular matrix structural constituent (GO:0005201); Complement activation, alternative pathway (GO:0006957); Chymotrypsin activity (GO:0004263); Cell adhesion (GO:0007155); Proteolysis and peptidolysis (GO:0006508); Calcium ion binding (GO:0005509); Catabolism (GO:0009056); Growth (GO:0040007); Trypsin activity (GO:0004295)
	Soluble secreted protein	None observed
Soluble intracellular and Type I membrane proteins	Soluble intracellular protein	Rhodopsin-like receptor activity (GO:0001584); Hematopoietin/interferon-class (D200-domain) cytokine receptor activity (GO:0004896); Cell surface receptor linked signal transduction (GO:0007166); Phosphoric monoester hydrolase activity (GO:0016791); Interleukin binding (GO:0019965); Phosphoprotein phosphatase activity (GO:0004721); Transmembrane receptor activity (GO:0004888); Protein amino acid dephosphorylation (GO:0006470); Dephosphorylation (GO:0016311); Phosphate metabolism (GO:0006796); Transmembrane receptor protein tyrosine kinase activity (GO:0004714); Cytokine binding (GO:0019955); Transmembrane receptor protein kinase activity (GO:0019199); Signal transduction (GO:0007165); Receptor activity (GO:0004872); Cell communication (GO:0007154); Phosphoric ester hydrolase activity (GO:0042578); Phosphorus metabolism (GO:0006793); Protein tyrosine phosphatase activity (GO:0004725); Transmembrane receptor protein tyrosine phosphatase signaling pathway (GO:0007185); Signal transducer activity (GO:000487); Interleukin receptor activity (GO:0004907); Enzyme linked receptor protein signaling pathway (GO:0007167)

Variable classes of protein orientation with respect to the membrane	Comparative category	Over-represented Gene Ontology terms
	Type I membrane protein	Signal transduction (GO:0007165); Phosphorus metabolism (GO:0006793); Cell surface receptor linked signal transduction (GO:0007166); Phosphoric ester hydrolase activity (GO:0042578); Protein tyrosine phosphatase activity (GO:0004725); Phosphoric monoester hydrolase activity (GO:0016791); Transmembrane receptor protein tyrosine phosphatase signaling pathway (GO:0007185); Hydrolase activity, acting on ester bonds (GO:0016788); Phosphoprotein phosphatase activity (GO:0004721); Protein amino acid dephosphorylation (GO:0006470); Dephosphorylation (GO:0016311); Phosphate metabolism (GO:0006796); Enzyme linked receptor protein signaling pathway (GO:0007167)
Soluble intracellular and Type II membrane proteins	Soluble intracellular protein	Carbohydrate binding (GO:0030246); Signal transducer activity (GO:000487); Receptor activity (GO:0004872); UDP-galactosyltransferase activity (GO:0035250); Sugar binding (GO:0005529); Galactosyltransferase activity (GO:0008378)
	Type II membrane protein	None observed
Soluble intracellular and Multi-span membrane proteins	Soluble intracellular protein	Transport (GO:0006810); Ion transporter activity (GO:0015075); Cation transport (GO:0006812); Transmembrane receptor protein tyrosine phosphatase signaling pathway (GO:0007185); Ion transport (GO:0006811); Carrier activity (GO:0005386); Localization (GO:0051179); Monovalent inorganic cation transport (GO:0015672); Antiporter activity (GO:0015297); Cation transporter activity (GO:0008324); Establishment of localization (GO:0051234)
	Multi-span membrane protein	None observed

Variable classes of protein orientation with respect to the membrane	Comparative category	Over-represented Gene Ontology terms
Soluble secreted and Type I membrane proteins	Soluble secreted protein	Phosphorylation (GO:0016310); Hematopoietin/interferon-class (D200-domain) cytokine receptor activity (GO:0004896); Phosphotransferase activity, alcohol group as acceptor (GO:0016773); Protein-tyrosine kinase activity (GO:0004713); Interleukin binding (GO:0019965); Protein binding (GO:0005515); Protein kinase activity (GO:0004672); Nucleotide binding (GO:0000166); Transmembrane receptor activity (GO:0004888); Phosphate metabolism (GO:0006796); Transmembrane receptor protein tyrosine kinase activity (GO:0004714); Cytokine binding (GO:0019955); Transmembrane receptor protein kinase activity (GO:0019199); Receptor activity (GO:0004872); Kinase activity (GO:0016301); Protein amino acid phosphorylation (GO:0006468); Cell communication (GO:0007154); Phosphorus metabolism (GO:0006793); Signal transducer activity (GO:000487); Protein serine/threonine kinase activity (GO:0004674); Protein modification (GO:0006464); Interleukin receptor activity (GO:0004907); Transferase activity, transferring phosphorus-containing groups (GO:0016772)
	Type I membrane protein	None observed
Soluble secreted and Type II membrane proteins	Soluble secreted protein	None observed
	Type II membrane protein	None observed

Variable classes of protein orientation with respect to the membrane	Comparative category	Over-represented Gene Ontology terms
Soluble secreted and Multi-span membrane proteins	Soluble secreted protein	Phosphorylation (GO:0016310); Hematopoietin/interferon-class (D200-domain) cytokine receptor activity (GO:0004896); Phosphotransferase activity, alcohol group as acceptor (GO:0016773); Protein-tyrosine kinase activity (GO:0004713); Interleukin binding (GO:0019965); Protein binding (GO:0005515); Protein kinase activity (GO:0004672); Nucleotide binding (GO:0000166); Transmembrane receptor activity (GO:0004888); Phosphate metabolism (GO:0006796); Transmembrane receptor protein tyrosine kinase activity (GO:0004714); Cytokine binding (GO:0019955); Transmembrane receptor protein kinase activity (GO:0019199); Receptor activity (GO:0004872); Kinase activity (GO:0016301); Protein amino acid phosphorylation (GO:0006468); Cell communication (GO:0007154); Phosphorus metabolism (GO:0006793); Signal transducer activity (GO:000487); Protein serine/threonine kinase activity (GO:0004674); Protein modification (GO:0006464); Interleukin receptor activity (GO:0004907); Transferase activity, transferring phosphorus-containing groups (GO:0016772)
	Multi-span membrane protein	Ligand-gated ion channel activity (GO:0015276); Excitatory extracellular ligand-gated ion channel activity (GO:0005231); Extracellular ligand-gated ion channel activity (GO:0005230); Transmission of nerve impulse (GO:0019226); Cell-cell signaling (GO:0007267); Glutamate receptor activity (GO:0008066); Synaptic transmission (GO:0007268); Ionotropic glutamate receptor activity (GO:0004970); Glutamate-gated ion channel activity (GO:0005234)
Type I membrane and Type II membrane proteins	Type I membrane protein	None observed
	Type II membrane protein	Signal transducer activity (GO:000487); Receptor activity (GO:0004872); Cell surface receptor linked signal transduction (GO:0007166); Phosphorus metabolism (GO:0006793); Phosphate metabolism (GO:0006796); Enzyme linked receptor protein signaling pathway (GO:0007167)
Type I membrane and Multispan membrane proteins	Type I membrane protein	None observed
	Multi-span membrane protein	None observed

Variable classes of protein orientation with respect to the membrane	Comparative category	Over-represented Gene Ontology terms
Type II membrane and Multispan membrane protein	Type II membrane protein	Carboxylic acid biosynthesis (GO:0046394); Organic acid biosynthesis (GO:0016053); Fatty acid biosynthesis (GO:0006633)
	Multi-span membrane protein	Synaptogenesis (GO:0007416); Extracellular matrix organization and biogenesis (GO:0030198); Synapse organization and biogenesis (GO:0050808); Extracellular structure organization and biogenesis (GO:0043062)
